# Dynamic randomization and a randomization model for clinical trials data

**DOI:** 10.1002/sim.5448

**Published:** 2012-07-05

**Authors:** Lee D Kaiser

**Affiliations:** Genentech, Inc.1 DNA Way, South San Francisco, 94080, CA, U.S.A.

**Keywords:** linear model, unit-treatment additivity, permuted-blocks randomization, biased-coin randomization, minimization, randomization test

## Abstract

Randomization models are useful in supporting the validity of linear model analyses applied to data from a clinical trial that employed randomization via permuted blocks. Here, a randomization model for clinical trials data with arbitrary randomization methodology is developed, with treatment effect estimators and standard error estimators valid from a randomization perspective. A central limit theorem for the treatment effect estimator is also derived. As with permuted-blocks randomization, a typical linear model analysis provides results similar to the randomization model results when, roughly, unit effects display no pattern over time. A key requirement for the randomization inference is that the unconditional probability that any patient receives active treatment is constant across patients**;** when this probability condition is violated, the treatment effect estimator is biased from a randomization perspective. Most randomization methods for balanced, 1 to 1, treatment allocation satisfy this condition. However, many dynamic randomization methods for planned unbalanced treatment allocation, like 2 to 1, do not satisfy this constant probability condition, and these methods should be avoided. Copyright © 2012 John Wiley & Sons, Ltd.

## 1. Introduction

A theory for estimation in a linear model, specifically accounting for the randomization used in a clinical trial will be developed. A particular goal is to compare the inferences from the usual population-based linear model with those from the randomization model. A further goal is to compare randomization model inference that follows from permuted-blocks randomization (PBR) with those from dynamic randomization methods. Here, ‘dynamic randomization’ refers to methodology in which the probability of assignment of a given patient to experimental treatment is a function of the patient's stratification variables and the stratification variables and treatment assignments of previously randomized patients. Other terminology has been used, such as adaptive randomization and minimization.

Randomization of treatments to patients in clinical trials has perhaps most commonly been performed with PBR, often within strata defined by crossings of prognostic factors and/or investigational sites. Permuted blocks are used instead of ‘complete randomization’, which assigns the next patient to experimental treatment essentially by a fair coin toss, to ensure that the number of patients assigned to each treatment group remains similar within strata, regardless of the number of patients ultimately enrolled in the strata [1]. Efron [2] introduced biased-coin randomization, which assigns the next patient to experimental treatment with a biased coin if the counts of patients previously assigned to the two treatments are imbalanced. Pocock and Simon [3] generalized the method to include stratification variables, where the probability of assignment to experimental treatment depends on the balance of treatment counts across the margins of the stratification variables. There have subsequently been many proposals for other dynamic randomization procedures [4–7].

A theory for estimation and testing exists for permutation-based randomizations paired with a linear model, where the linear model includes the randomization's stratification variables in addition to a treatment term, and leads to unbiased estimators of treatment effects and unbiased estimators of the variance of the treatment effect estimator [8–11]. The unbiasedness is over the distribution of the observations induced by repeated randomizations of treatments to fixed experimental units, under the assumption of unit-treatment additivity with fixed unit and treatment effects.

There have been many theoretical developments for randomizations that are not of the permutation type, but these have mostly focused on randomization tests rather than estimation.

◦ Smythe and Wei [12] derived the asymptotic distribution of a linear rank test of the null hypothesis of equality of two treatments when the assignment of treatments to patients is according to an urn-based randomization scheme.
◦ Smith [13] summarized treatment allocation methods developed to date. For treatment allocation probabilities that are functions of (*n*_1_ − *n*_0_) / (*n*_0_ + *n*_1_), where the *n*_*j*_'s are the numbers of patients previously assigned to the two treatments, Smith developed an approximation to a randomization test based on the difference in sample means. Smith also developed similar approximations when treatment assignment probabilities are a particular function of stratification variables of the current and previous patients and developed asymptotic approximations to randomization tests.
◦ Wei *et al.* [14] proposed a biased-coin scheme for the allocation of *k* treatments in proportion ξ_1_, …, ξ_*k*_, where ξ_1_ + … + ξ_*k*_ = 1, and generalized the work of Smythe and Wei [12] to *k* > 2.
◦ Shao *et al.* [15] proposed a theory for clinical trials with dynamic randomization, but the results were developed on an assumption of a model that relates the dependent variable to the variables used in constructing hypothesis tests.
◦ Rosenkranz [16] explored the sampling properties of a difference in sample means under various randomization schemes and noted that a *t*-test can be very conservative with biased- coin randomization.

Regardless of the randomization method used, it is common in clinical trials data analysis to apply a statistical test that is based on assumptions of random sampling from a distribution. In general, these tests behave as follows for biased-coin and urn-based randomizations [2, 13, 16–18].

◦ If there is low-frequency variation in the responses of patients over time of entry into the trial, then a population-based test will tend to result in a larger *p*-value than the corresponding randomization test result. With this pattern of variation, patients enrolled closer together in time tend to respond more similarly than patients enrolled farther apart in time.
◦ If there is high-frequency variation in responses over enrollment time, then a population-based test will tend to yield a smaller *p*-value than the corresponding randomization test result. In this case, patients enrolled closer together in time tend to respond less similarly than patients enrolled farther apart in time. This is generally noted to be less likely than the scenario above.
◦ Finally, when responses appear to come from a homogeneous, time-independent model, then the two test results tend to be similar.

These same qualitative comparisons apply for PBR when the blocking in time is ignored in the analysis [1], in which case the variation in patient responses is quantified by the intrablock correlation.

Motivation for the present work on estimation and testing under a randomization model comes from a ‘points to consider’ document on adjustment for baseline covariates [19] that states ‘… techniques of dynamic allocation such as minimization are sometimes used to achieve balance across several factors simultaneously. Even if deterministic schemes are avoided, such methods remain highly controversial. Thus, applicants are strongly advised to avoid such methods.’ Furthermore, Halpern and Brown [18] noted that dynamic randomization schemes ‘can lead to complex randomization distributions and hence to complex analyses about which little is currently known, and which, in fact, may present intractable difficulties.’ With a randomization-based theory for estimation and testing applicable across all randomization methods, perhaps regulatory authorities will be more accepting of dynamic randomization methods.

Section 2 contains the theory for randomization-based estimation in a linear model with unit-treatment additivity. Section 3 evaluates the estimation of the variance of the treatment effect estimator through a population-based linear model, in comparison with the randomization-based variance estimator. Section 4 compares the estimation results with randomization test results. Sections 5 through 7 explore randomization-based inference when there is planned unbalanced treatment allocation, like two experimental to one control. A key requirement, often not met with proposed dynamic randomization methods for such unbalanced allocation, is that the probability that a patient receives experimental treatment should be constant across patients. The paper finishes with a discussion and conclusion.

## 2. The randomization model

Suppose *n* patients are in the trial, and they are assigned one of two treatments: experimental or control. The probability that a patient receives experimental treatment is a function of this patient's stratification variables and the stratification variables and treatment assignments of previous patients. No assumptions are made on this function just yet. Let **δ** be the *n* × 1 vector that indicates assignment to experimental treatment: δ_*i*_ = 0 if patient *i* receives control and 1 if patient *i* receives experimental treatment. Assume unit-treatment additivity, so that **y**, the *n* × 1 vector of observations on the patients, is



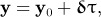


for unknown constant unit effects **y**_0_ and a scalar treatment effect τ. A vector of independent and identically distributed random variables could be added as an error term to the model, but this only complicates the development without changing the conclusions.

Let **X** be an *n* × *q* matrix of covariates measured on the patients. Typically, some or all of the stratification variables used in the randomization will be included in **X**, but this is not required in what follows. Also, it will typically be thought that the variables in **X** are prognostic of patient outcome, but there is no assumed relationship between the unit effects in **y**_0_ and the covariates in **X**. The only requirement is that **1**, an *n* × 1 vector of ones, is in the column space of **X** and, without loss of generality, **X** has full column rank. As a simple example, **X** could have two columns, with the first column containing 1s and the second column denoting Eastern Cooperative Oncology Group performance status of 1 or more. An analysis of covariance example has **X** with a column of 1s and a column for a continuous covariate.

We want to estimate τ after adjustment for **X**, and so use the normal equations


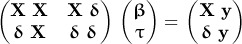


where ***β*** is a vector of nuisance parameters. With simple matrix algebra,


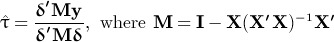


It is worthwhile to explicitly state what is fixed and what is random in this set-up, because this will provide the relative frequency basis for the inferences. The patients have entered the study in a given order, and while they could conceptually have entered in a different order, it is not clear what a reasonable probability model should be for these other potential patient entry orders. Also, would the patients’ unit effects be different if they entered in a different order, and how should these effects be modeled? Would the patients’ baseline covariates differ? Thus, patient entry order, the unknown unit effects, and patient covariates are considered fixed. What does clearly vary, and in a way that we can model, is the treatment assignment to patients, as captured in **δ**. The variability in the observations **y** then comes only from the randomization mechanism, and the conceptual repetitions of the study arise from independent applications of the randomization method to these patients as entered with their fixed covariates and unit effects. This topic will be addressed further in the discussion section.

Up to this point, no assumptions have been made on the randomization algorithm, and the only one needed to make progress is that *E***δ** = *p***1**, with *p* a constant: 1 to1 randomization would have *p* = 0.5, 2 to 1 randomization would have *p* = 2 / 3, and so on. The problems with randomization methods with *E***δ** ≠ *p***1** are addressed in Section 6.

This restriction to *E***δ** = *p***1** means that the unconditional probability that each patient receives experimental treatment is constant, even though the probability that a patient receives experimental treatment, conditional on the treatment allocations of previous patients, will often be different from *p* to more closely attain treatment balance, either overall or within stratification variable groupings. Note that it is not enough that *E***δ** averages to the desired fraction, like (1 / *n*)**1**^***′***^*E***δ** = 1 / 2. With 1 to 1 treatment allocation, the *E***δ** = (1 / 2)**1** condition is easily satisfied because of the usual symmetry present in randomization algorithms, while dynamic randomization algorithms for unbalanced treatment allocations often do not maintain a constant *E*δ_*i*_ value [20]. Examples of this are given in Section 6.1 and a potential solution for certain cases is given in Section 6.

Note that 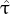 is a function of **δ**^***′***^**Mδ**, which does not depend on unknown parameters and so is an ancillary statistic. In simple cases, as in Table I, ***δ′*Mδ** is a function of sample sizes within the strata, and most would prefer to condition on observed sample sizes [21]. As shown in Appendix B, **δ**^***′***^**Mδ** / *n* converges in probability to *p*(1 − *p*) under mild conditions, so for large samples, inference that is conditional on ***δ′*Mδ** and unconditional inference should be similar. However, for small samples, conditional inference may be preferred, so both approaches are developed in the next sections.

**Table I:** *δ′*Mδ for three examples of the X matrix

X	δ′Mδ
Intercept term only	(1 / *n*_0_ + 1 / *n*_1_)^−1^, where *n*_*j*_ is the number of patients on treatment *j*
Two columns denoting membership in one of two strata, such as low ECOG status vs. high status	(1 / *n*_10_ + 1 / *n*_11_)^−1^ + (1 / *n*_20_ + 1 / *n*_21_)^−1^, where *n*_*ij*_ is the number of patients on treatment *j* in stratum *i*
Intercept and continuous covariate as in an ANCOVA	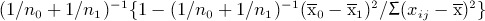 , where *n*_*j*_ is the number of patients on treatment *j*, *x*_*ij*_ is the covariate value of patient *j* on treatment *i*

### 2.1. Unconditional inference

Appendix A.1 develops approximations to the mean and variance of 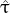 under the restrictions of *E***δ** = *p***1** and unit-treatment additivity and without conditioning on a given **δ′Mδ** value. First, 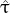 is an approximately unbiased estimator of τ. Second,


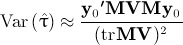
(1)

where tr() denotes the trace of a matrix and **V** = Var(**δ**) is the variance–covariance matrix of **δ**. 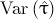 is a function of the residuals in the regression of **y**_0_ on **X**, so good use of covariance adjustment reduces the variance, which is as it should be. It is not so clear what the role of the randomization method is in the reduction of variance, because it is tied up in the **V** matrix. As an example, however, especially in a small study with a stratified analysis, a stratified permuted-blocks randomization is better than unstratified permuted-blocks randomization [22, 23].

Next, it is shown in Appendix A.1 that


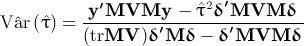
(2)

is an approximately unbiased estimator of the 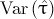 approximation of Equation (1). Finally, under mild conditions it is shown in Appendix B that 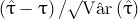 has asymptotically a standard normal distribution, so that tests and confidence intervals on τ can be determined in the usual way.

In a common class of randomization methods, 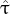 is unbiased, and not just approximately unbiased. Define a symmetrical 1 to 1 randomization method as one with *P*(**δ** = ***δ***_0_) = *P*(**δ** = **1** − **δ**_0_) for each treatment assignment sequence **δ**_0_. In words, the probability of obtaining any treatment sequence equals the probability of that sequence with the treatments switched between experimental and control. Most 1 to 1 randomization methods treat the experimental and control arms symmetrically and satisfy this condition. For such randomization methods, the unbiasedness of 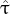 is shown in Appendix A.1.

The diagonal elements of **V** are *p*(1 − *p*) and the off-diagonal elements are *E*(δ_*i*_δ_*j*_) − *p*^2^, where *E*(δ_*i*_δ_*j*_) is the probability that patients *i* and *j* both receive experimental treatment. Analytical expressions for the first two moments of counts of assignments to treatments for urn-based randomizations [24] and for biased-coin randomizations [25] are available. For some cases, *E*(δ_*i*_δ_*j*_) can be determined algorithmically by stepping through all assignment probabilities [26]. Alternatively, a straightforward solution for any randomization method is to rerandomize treatments to the patients in the trial according to the trial's algorithm millions of times and estimate **V** with the sample variance–covariance matrix of the resulting **δ**s.

### 2.2. Conditional inference

For symmetrical 1 to 1 randomizations as defined above, inference conditional on a **δ′Mδ** value is developed in Appendix B.1, where first it is shown that 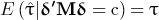. Furthermore, letting 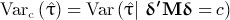, it is shown that


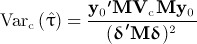


where 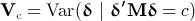. Finally,


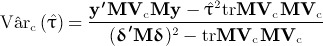


is a conditionally unbiased estimator of 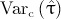.

Because most 1 to 1 randomizations are symmetrical, conditional inference is straightforward, as outlined above. For planned unbalanced allocation, however, even when *E***δ** = *p***1**, *E*(δ_*i*_ | **δ′Mδ** = *c*) is typically not constant over *i*. For example, consider PBR in blocks of 6 in a 2 to 1 ratio to 10 patients, with **M** = **I** − **J** / 12. Unconditionally, *E*δ_*i*_ = 2 / 3. However, from Table I, if **δ′Mδ** = (1 / 8 + 1 / 2)^−1^ = 1.6, then *E*(δ_*i*_ | **δ′Mδ** = 1.6) = 2 / 3 for 1 *≤ i ≤*6, but E(δ_*i*_ | **δ′Mδ** = 1.6) = 1 for 7 *≤ i ≤*10.

The variation in the conditional probability of assignment to experimental treatment leads to conditional bias in 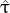. Let 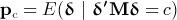, then


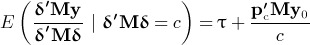


and the conditional bias is proportional to the covariance between **p**_c_ and **My**_0_. The problem of conditional inference for planned unbalanced allocations with *E***δ** = *p***1** is illustrated in Section 5 and a potential solution is raised in the conclusion section.

## 3. Comparison of standard error estimators — linear model versus randomization model

Regardless of the randomization method used, it is common to analyze trial data with a population-based linear model


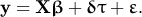
(3)

The elements of ***ϵ*** are independent and identically distributed with zero mean and variance σ^2^, **δ** is considered fixed, and **X** often includes stratification factors that are balanced by the randomization [27]. It is common, however, to ignore in the model the balancing in time that randomizations typically achieve.

By construction, the treatment effect estimator is the same under the randomization model as under model (3); however, the variance estimator from Equation (2) will generally differ from the variance estimator derived from model (3). How similar are these estimators from a randomization perspective? The estimators will undoubtedly be different in any particular trial, but it is informative to consider their expectations.

The linear model estimate of the variance of 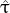 is


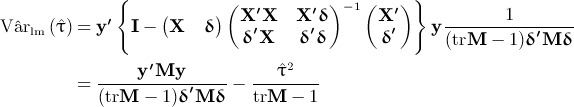
(4)

What is the expectation of 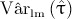 from a randomization perspective? Again, the numerator and denominator both contain random variables, but approximate by simply taking expectations individually of the numerator and denominator to obtain


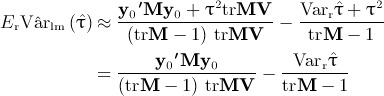
(5)

where the subscript r on *E*_r_ and Var_r_ denotes expectation and variance over the randomization method used. For what unit effects **y**_0_ might 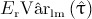 be similar to, less than, or greater than 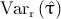
? Some guidance is given in the next two sections.

### 3.1. Linear model versus randomization model standard error estimators when no pattern is expected for unit effects

Suppose that apart from dependence on **X**, the unit effects **y**_0_ display no further systematic variation. A reasonable model for this is **y**_0_ = **Xβ** + **e**, where **e** is a vector of independent and identically distributed random variables with expectation zero and finite variance σ^2^. It is easy to see that the expectation of both 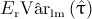 and 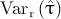 equals σ^2^ / tr**MV**.

Therefore, if no discernable pattern in unit effects is expected, apart from variation owing to dependence on **X**, then the usual linear model estimate of the estimated treatment effect variance estimates a quantity similar to the proper randomization variance. This is true regardless of the type of randomization, as long as *E***δ** = *p***1**.

Note also that if complete randomization were used, with **V** = *p*(1 − *p*)**I**, then the randomization model variance estimator in (2) equals the linear model estimator in (4), illustrating the equivalence of randomization-based and population-based inference with complete randomization [28].

### 3.2. Linear model versus randomization model standard error estimators when unit effects have systematic variation

The difference between the expectation of the linear model variance estimator in Equation (5) and the randomization variance in Equation (1) is proportional to 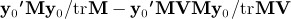. These quadratic forms have been evaluated in the context of accidental bias by Efron [2] and Smith [13] for biased-coin randomization.

Efron defined accidental bias as the expectation, over the randomization used, of the squared bias of 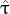 calculated under an assumed regression model for the observations, where the regression model includes a covariate **z** not accounted for in the treatment effect estimator. This squared bias is proportional to **z′MVMz**, and therefore the work of these authors applies to the comparison of variance estimators considered here. The applicable conclusion for biased-coin randomization is that when unit effect residuals, after regression on **X**, exhibit low-frequency variation or a smooth trend from patient to patient, then the linear model estimate of variance will tend to be larger than the randomization model estimate. With high-frequency variation, the comparison of the variances is reversed.

Urn randomization is asymptotically free of accidental bias [17] so that for very large samples, the linear model variance estimate is likely to be similar to the randomization-based variance. For smaller samples, the control of sample size differences achieved by urn randomization is likely to lead to comparisons of variances similar to those for biased-coin randomization.

This parallels the situation with PBR where the variance estimate ignoring the blocking in time will generally be greater than the estimate accounting for this blocking when there is low-frequency variation or a smooth time trend in the unit effect residuals, as quantified by the intrablock correlation [1].

## 4. Randomization tests

The null hypothesis that treatment has absolutely no effect in any patient can be tested with a randomization test. Under this hypothesis, the vector of observations **y** equals **y**_0_, and the observed 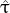 is just one of the possible treatment effect estimates that could be obtained by the random assignment of treatment labels to these patients. Is the observed 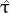 unusually large or small in this distribution of possible 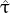 values?

A randomization test is implemented by performing the randomization repeatedly with the patients enrolled in their fixed order and determining the resulting 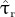 values. For an alternative hypothesis of positive τ, the randomization test *p*-value is estimated as the fraction of the 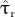 values that are greater than the observed 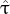; for a two-sided alternative, compare 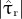 to 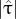. As with estimation, the randomization test can be performed conditionally on **δ′Mδ** = *c* or unconditionally. For the conditional randomization test, only those randomizations with **δ′Mδ** = *c*, or suitably close to it, are used for the generation of the 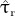 values.

What are the first two moments of this randomization distribution of 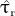
? With *E***δ** = *p***1**, from Section 2.1, unconditionally 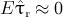 and


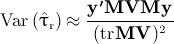
(6)

Under the asymptotic normality conditions at the beginning of Appendix B, an approximation to the randomization test would be to compare 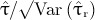 with standard normal quantiles. This test statistic is similar to the *Z*-statistic obtained from use of 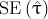 from Equation (2), except that a ‘sum of squares for treatment’ is removed in (2) relative to the variance in the randomization distribution. Through use of approximations as in Appendix A.1, the expectation of Equation (6) exceeds that of Equation (2) by approximately τ^2^tr**MVMV** / (tr**MV**)^2^, which is small for small τ or large *n*.

Thus, the randomization test inference will necessarily be similar to the inference that comes from simple use of 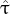 and its estimated standard error. As above, however, if the estimated standard error is obtained from a linear model based on **X**, then the randomization test need not agree with the linear model results. From Section 3.1, when the unit effects display no trend apart from dependence on **X** then the randomization inference should be similar to that from linear model inference. Likewise, from Section 3.2, when unit effects display a low-frequency trend, then the randomization test *p*-value would be expected to be smaller than the *p*-value from the linear model, with the reverse order when the unit effects display high-frequency variation. This agrees with the summary of randomization tests given in the introduction.

## 5. Conditional inference with planned unbalanced treatment allocation ratio

Section 2.2 noted that planned unbalanced treatment allocation with *E***δ** = *p***1** yields an approximately unbiased treatment effect estimator unconditionally, but the estimator is typically biased conditioned on a **δ′Mδ** value. This section illustrates by example the potential for bias with conditional inference and shows that the problem is applicable to PBR and to dynamic randomization.

Consider the randomization of two treatments to 38 patients in a 2 to 1 ratio, with patients categorized by two binary stratification variables. The patient counts in the four cells defined by these two variables are respectively 10, 8, 11, and 9 for cells (0,0), (0,1), (1,0), and (1,1), with the first value of the pair indicating the Stratum 1 level and the second value indicating the Stratum 2 level. The order of entry of these patients to the simulated trial was determined at random and then held fixed.

Two randomization methods are evaluated. The first is stratified PBR, with 2 to 1 allocation in blocks of six applied independently within each of the four cells. The second is a sequential method that follows the recommendation at the end of Section 6.1 to perform a symmetrical 1 to 1 to 1 randomization with three treatments, with two treatment arms subsequently combined (see Appendix C for details). The matrix **X** includes an intercept and main effects for the two strata. Each randomization method was replicated 10 million times to determine the **V**_c_ matrices for this population. The methods were subsequently replicated one million times to determine the conditional properties of the estimators.

**Figure 1:**
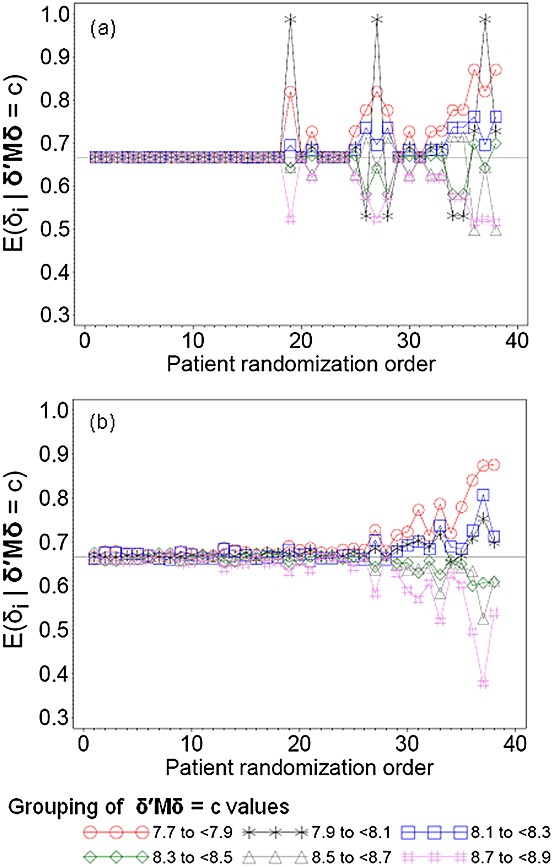
E(δi | δ′Mδ = c) versus patient randomization order, with values of c grouped to the nearest 0.2. (a) Stratified permuted-blocks randomization and (b) sequential randomization. See text for details on these randomization methods.

For each randomization method, the expected value of the ancillary statistic **δ′Mδ** is 8.3 and approximately 90% of the **δ′Mδ** values are between 7.7 and 8.9; smaller values represent randomizations that generally exceed the 2 to 1 ratio and larger values represent randomizations with closer to 1 to 1 allocation. Figure 1 illustrates the fluctuation across the patients in the empirically estimated *E*(δ_*i*_ | **δ′Mδ** = *c*) for selected values of *c*, grouped to the nearest 0.2. For stratified PBR, *E*(δ_*i*_ | **δ′Mδ** = *c*) is exactly 2/3 for earlier patients who belong to complete blocks, and similarly for the sequential method, *E*(δ_*i*_ | **δ′Mδ** = *c*) is approximately 2/3 for early patients. However, for patients in incomplete blocks with PBR and for the later patients with the sequential method, the conditional probability of assignment to active treatment diverges from 2/3, with *E*(δ_*i*_ | **δ′Mδ** = *c*) generally above 2/3 for smaller values of *c* and below 2/3 for larger values of *c*.

**Figure 2:**
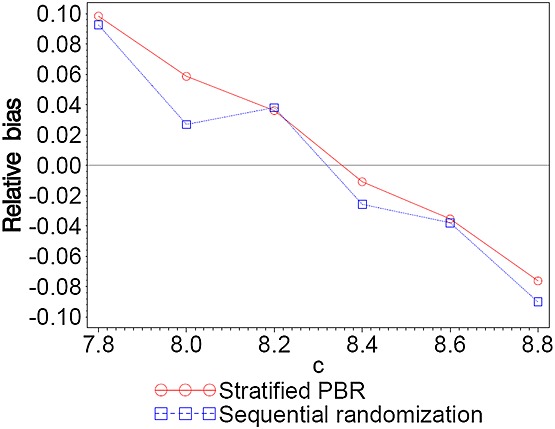
Conditional relative bias of 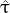 versus values of δ′Mδ = c grouped to the nearest 0.2. Conditional relative bias is the conditional bias of 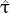 divided by the square root of the conditional variance of 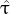. See text for details on the stratified permuted-blocks randomization and sequential randomization methods.

From Section 2.2, the conditional bias is proportional to the covariance between 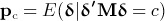 and the residuals of the unit effects after regression on **X**. Suppose here that unit effects are related to order of patient entry via a simple linear regression with a positive slope and coefficient of determination *R*^2^, and let *E*_lm_ denote the expectation with respect to this model. Through simple algebra, the expectation of the conditional bias normalized by 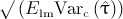 is


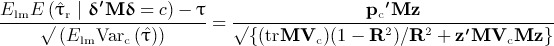


where **z** is a unit-length vector proportional to patient randomization order. This normalized bias is displayed in Figure 2 for each randomization method for *R*^2^ = 0.5 and **X** = **1**. The normalized bias is similar between randomization methods and is positive for smaller values of **δ′Mδ** and negative for larger values of **δ′Mδ**. At the smallest and largest values of **δ′Mδ** displayed the normalized bias is approximately 10% in absolute value.

Thus, for planned unbalanced treatment allocation with *E***δ** = *p***1**, randomization inference conditional on **δ′Mδ** can be problematic, and this problem is present with both dynamic randomization and PBR. Because this problem arises in stratified PBR only for the patients in the last potentially incomplete block in each stratum, the bias is likely negligible in large trials. Likewise, for dynamic randomization, although based on limited empirical evaluations, **p**_c_ appears to diverge from the desired fraction only for the latest patients randomized in a trial, so that the conditional bias for large trials would also seem to be negligible.

The next sections shift to the problem of unconditional bias when *E***δ** ≠ *p***1** and illustrate that this problem is common with planned unbalanced treatment allocation.

## 6. Estimation and randomization tests when *E*δ ≠ *p*1

When *E***δ** = **p** ≠ *p***1**, then 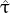 is biased. Using a first-order approximation, the bias is


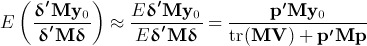


The bias is a function of the correlation between **p** and the residuals from the regression of **y**_0_ on **X**.

For the randomization test when *E***δ** ≠ *p***1**, the randomization distribution of the usual estimator 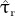 is not centered at zero, but at 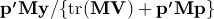, approximately. This nonzero expectation does not affect the Type I error rate by construction of the randomization test, although the power of the test may be affected [30].

A notable example of *E***δ** ≠ *p***1** is contained in the documents of the Endocrinologic and Metabolic Drugs Advisory Committee meeting held October 21, 2008 [30, 31]. In this case, the linear model inference indicated a statistically significant treatment effect, while the randomization test did not, resulting in *p* = 0.035 versus *p* = 0.06 for the analysis of one endpoint and *p* = 0.046 versus *p* = 0.15 for another endpoint.

Because the bias is a function of **p′My**_0_, a simple method to obtain an approximately unbiased estimator of τ is to augment **X** with the vector **p**, use [**Xp**] in the normal equations, and proceed as before with estimation of τ and the standard error. A randomization test would also proceed as before. The asymptotic normality results of Appendix B were developed with *E***δ** = *p***1**, so it is uncertain whether asymptotic normality applies in this case. Even if it did, the best solution is not to use randomization methods with *E***δ** ≠ *p***1** in the first place.

### 6.1. Dynamic randomization with planned unbalanced treatment allocation ratio

Consider randomizations for a trial with 2 to 1 randomization of experimental to control treatment. *P*(δ_*i*_ = 1) = 2 / 3 for PBR applied in blocks of multiples of three. What about biased-coin randomization? A natural attempt is to use a biased coin whenever *n*_e_ / 2 differs too much from *n*_c_, where *n*_e_ and *n*_c_ denote the number of patients previously assigned to experimental and control. Suppose a threshold of 1 or more is used, with *P*(δ_*i*_ = 1) = 0.9 for too many previous control arm assignments, *P*(δ_*i*_ = 1) = 0.2 for too many previous experimental arm assignments, and *P*(δ_*i*_ = 1) = 2 / 3 otherwise. This procedure satisfies conditions C1 and C2 in [13] and the probabilities satisfy the recent recommendation of Han *et al.* [6]. With the first patient's assignment to experimental treatment determined with probability 2/3, it is easy to calculate the *P*(δ_*i*_ = 1) values for *i* = 2, 3, 4, and 5 as 0.744, 0.467, 0.674, and 0.761. Stepping through the probabilities algorithmically yields *P*(δ_20_ = 1) = 0.775. The probabilities do not converge to 0.667, although the average of these 20 assignment probabilities is close to 0.667.

The situation is worse with the marginal balance metric [6]. In this simple case, the biased coin is applied to the treatment that yields the smaller value of 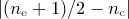 versus 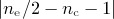. If the former is smaller, then experimental treatment is assigned with probability 0.9, and if the latter is smaller, then experimental treatment is assigned with probability 0.2. With equal values and also for the first patient, experimental treatment is assigned with probability 2/3. *P*(δ_*i*_ = 1) values for *i* = 2, 3, 4, and 5 are 0.433, 0.807, 0.881, and 0.342. Stepping through the probabilities algorithmically yields *P*(δ_20_ = 1) = 0.340.

The generalization of 1 to 1 urn randomization to unbalanced allocation in Rosenberger and Lachin [32] is incorrect. For example, with 2 to 1 allocation, and starting with 2 E balls and 1 C ball, one application of their generalization would add 2 E balls if control treatment is assigned, with 1 C ball added if experimental treatment is assigned. The first patient is assigned experimental treatment with probability 2/3, and *P*(δ_*i*_ = 1) values for *i* = 2, 3, 4, and 5 are 0.6, 0.590, 0.589, and 0.588. These probabilities converge to 0.586. A fix in this simple case is to add 4 E balls, rather than 2 E balls, if control treatment is assigned, with other aspects of the method unchanged. Then the *P*(δ_*i*_ = 1) values for *i* = 2, 3, 4, and 5 are 0.620, 0.641, 0.656, and 0.662 and the probabilities converge to 2/3. In large samples, urn sampling behaves like complete randomization [17] so that deviation from the target allocation ratio is likely to be greater with urn sampling than with other methods.

A solution for *r*_1_ : *r*_2_ treatment allocation is to perform randomization for balanced allocation to *r*_1_ + *r*_2_ treatment arms, and then combine *r*_1_ of these arms for the experimental arm assignment and *r*_2_ of these arms for control. As long as the randomization method treats the *r*_1_ + *r*_2_ arms symmetrically then *E***δ** = *p***1** will hold, and unconditional inference can be carried out as developed above.

## 7. Discussion

The estimation of τ involves only **δ**, **M**, and **y**, and the estimator 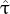 is approximately unbiased as long as *E***δ** = *p***1** and unit-treatment additivity holds. This approximate unbiasedness is true regardless of the relationship between **y**_0_ and **X** and any other properties of the randomization algorithm. Therefore, at least from the point of view of unbiased estimation of τ, there is no natural determination of **X** based on the randomization used, such as if the randomization is stratified on a particular factor, then that factor should be included in **X**. However, from the point of view of 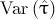, covariates that are related to the unit effects should be included in **X**, as smaller residuals from a regression of **y**_0_ on **X** reduce 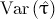. It is unclear how manipulation of the randomization scheme influences 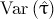, but it is likely that randomization methods that seek to balance the covariates in **X** between treatment groups are somewhat better than those that do not, at least for small studies. Finally, for a proper randomization-based variance estimator, both **X** and the randomization method, through **V**, should be accounted for. On this point, the advice to ‘analyze as you randomize’ should be followed.

Variability in the observations in the randomization model arises only from the random assignment of treatments to the patients in the order they entered the trial. This order and the patients’ baseline covariates determine the distribution of the treatment assignment vector **δ** and subsequent properties of the treatment effect estimator. In the discussion of randomization tests, this fixing of patient order is common [12, 13, 17, 26, 33]. A reviewer asked whether study conclusions can only be applied to these patients with the same order of entry? Now, it is common [34, 35] to consider that statistical inference in a randomized trial applies to the actual patients in the trial, with inference to the broader population of similar patients being either nonstatistical or based on assumptions that trial data follow specified distributions. In answer to the reviewer's question, it seems necessary to go a bit further and to say that the statistical inference from the randomization model applies only to these patients as they entered, at their specific stage of disease, and as treated at their specific study site. It is certainly plausible that treatment would have exerted a similar effect if patients had enrolled in a different order and under somewhat different circumstances, but to make statistical statements about such a treatment effect would require further assumptions.

One generalization of the model of Section 2 is to drop the assumption of unit-treatment additivity. Let τ_*i*_ be the difference in response if patient *i* were given experimental treatment versus the patient's response with control treatment. The estimator 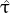 in Section 2 is in general a biased estimator of 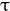, defined as the average of the τ_*i*_ values across the *n* patients. Using the first-order approximation to the expectation of a ratio of random variables as in Section 2.1, the bias is a function of a contrast in the diagonal elements of the matrix **VM**. These diagonal elements will often be nearly constant, in which case the bias will be small. Gadbury [36] showed for permutation-based randomizations that the usual estimator of 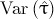 is positively biased. If a similar result applied to the variance estimator in Equation (2) for the more general randomizations considered here, then the randomization model estimators would yield, for example, nearly unbiased estimates of the average treatment effect, along with a conservative standard error estimate and conservative hypothesis tests on 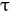.

Another generalization is to more than two treatments. This appears to be relatively straightforward for a continuous endpoint with unit-treatment additivity through the introduction of a separate **δ** vector for each of *t* − 1 treatments.

It would be interesting to see whether randomization model inference could be extended from the continuous endpoints considered here to binary responses and time-to-event endpoints. Certainly, randomization tests of a null hypothesis of no treatment effect in any patient are available for such endpoints, but treatment effect estimation from a randomization perspective does not seem to be available.

In addition to the analysis of the whole trial, it is important to explore treatment effects within subgroups of patients. If *E***δ** = *p***1** and there is unit-treatment additivity within the subgroup, then a linear model estimate within the subgroup of this common treatment effect as in Section 2 is approximately unbiased, regardless of the covariates included in such an estimate. An estimate of the standard error of this estimator could come from Equation (2), with **V** determined only from this subset of patients. As in Section 3.1, if unit effects within this subset are expected to display no systematic variation other than dependence on the covariates, then the linear model estimate of the standard error should be a reasonable approximation to the randomization-based standard error estimate.

## 8. Conclusion

For symmetrical 1 to 1 randomization, inference conditional on **δ′Mδ** and unconditional inference are both straightforward, and a randomization test will necessarily be similar to a test result obtained from 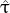 and its estimated variance, which is justified by a central limit theorem. With planned unbalanced treatment allocation, Proschan *et al.* [30] argued to ‘minimize the use of minimization.’ However, it is only with *E***δ** ≠ *p***1** that unconditional inference is problematic. Unfortunately, as illustrated in Section 6.1, many dynamic randomization methods for planned unbalanced allocation result in *E***δ** ≠ *p***1**, and care must be exercised to achieve *E***δ** = *p***1**, in which case unconditional inference is also straightforward. Although likely only relevant for smaller trials, with *E***δ** = *p***1** and planned unbalanced allocation, conditional inference will typically lead to bias in 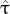. This conditional bias is also present with PBR when the blocking in time is ignored in the analysis. A potential repair to 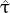 is to include, as suggested in Section 6 for unconditional inference, 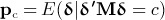 as a covariate in **X** and proceed with inference based on 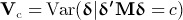. This possible repair needs further evaluation.

An additional similarity between PBR and dynamic randomization methods that attempt to balance treatment counts is that inference from a population-based model will likely be similar to a randomization-based analysis when unit effects, or rather the residuals from the regression of unit effects on **X**, are essentially sampled from a homogenous population. Furthermore, for both PBR and dynamic methods, when unit effect residuals display low-frequency variation or a general trend related to enrollment time, then the estimated variance of 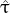 from a population-based model will tend to overestimate the randomization-based variance, with the opposite order in variance terms for high-frequency variation.

In our company's experience, the use of dynamic randomization has never been an issue in disqualifying a positive study. However, at the time of protocol review, we often receive comments from health authority review staff on our randomization method and trial analysis methods. It is very common that we receive a request for a randomization test for the main trial endpoints. I believe we would not receive such comments and requests if we routinely used PBR. With the results of this manuscript, owing to the similarity between randomization inference for dynamic randomization and PBR, it seems inconsistent to single out dynamic methods for the performance of a randomization test. Given that the blocking in time achieved by PBR and dynamic methods is routinely ignored in analyses, one should either (1) be satisfied that these analyses are likely to match a randomization analysis or to be conservative or (2) ask for a randomization test for any randomization method other than complete randomization.

## Appendix A.1

Approximations to the unconditional moments of  will be developed here under the assumptions of *E***δ** = *p***1** and unit-treatment additivity. Recall that **X** is an *n* × *q* full column rank matrix with **1** in its column space and that **M** = **I** − **X**(**X′X**)^−1^**X**. First, note that

so that the moments of  can be found from the moments of . Approximate this ratio with a first-degree Taylor series expansion about the means of the numerator and denominator to obtain the approximation **δ′My**_0_ / tr**M****V**, where **V** = Var(**δ**) and tr() denotes the trace of a matrix. Thus,

because **1** is in the column space of **X**, so that . Therefore,  is approximately unbiased.

For the variance of ,

For the estimation of , note that

Define

It is easy to see that

Because ,  is an approximately unbiased estimator of .

A problem with , inherent in its unconditional nature, is that it averages over variation in **δ′Mδ**. A potentially better estimator that explicitly depends on **δ′Mδ** can be found by requiring that the variance estimator match the linear model estimator of Equation (4) in Section 3.0 when complete randomization with **V** = *p*(1 − *p*)**I** is used. With this requirement

Approximating the expectation of the ratio by the ratio of the expectations and approximating  by the product of the expectations,  is also an approximately unbiased estimator of .

Appendix A.2 shows for symmetrical 1 to 1 randomization methods, defined in Section 2.1, that  is unbiased conditional on **δ**^***′***^**M****δ** = *c*. The estimator  is therefore unconditionally unbiased for such randomization methods.

## Appendix A.2

For the properties of  conditional on **δ′Mδ** = *c*, it is first necessary to evaluate *E*(**δ** | **δ′Mδ** = *c*). In general, this conditional expectation is not proportional to the vector **1** even with *E*(**δ**) = *p***1**. An example for PBR in blocks of six for treatments in a 2 to 1 ratio is given in Section 2.2.

In spite of potential bias issues with conditional inference with planned unbalanced treatment allocation,  is conditionally unbiased with symmetrical 1 to 1 randomization, defined in Section 2.1, where *P*(**δ** = ***δ***_0_) = *P*(**δ** = **1** − **δ**_0_). To show this, note first that if **δ′Mδ** = *c* then (**1** − **δ**)**′****M**(**1** − **δ**) = *c*. Thus, let {**δ**_*ij*_} denote all treatment assignment sequences with , where **δ**_*i*2_ = **1** − **δ**_*i*1_. Also, let *p*_*ij*_ denote the probability of obtaining the sequence **δ**_*ij*_. Because of symmetry, *p*_*i*1_ = *p*_*i*2_. By definition,

Hence,  for all *c*.

Let  denote . Continuing with symmetrical 1 to 1 randomization, it is easy to see that

where . Following the approach in Appendix A.1,

is a conditionally unbiased estimator of .

## Appendix B

Here, a proof is sketched of the asymptotic normality of  under the randomization model with *E***δ** = *p***1**. As in Section 2,  is adjusted for **X**, which has full column rank *q* and **1** in its column space.

Intuitively, for reasonable randomization algorithms, treatment assignments for very large *n* have almost no dependence on treatment assignments for small *n*, so central limit theorems for sequences of random variables that satisfy a ‘strong mixing’ condition [37, 38] should be helpful. However, the strong mixing conditions are difficult to verify for randomization algorithms.

The approach taken here, then, is to approximate the sequence δ_1_,δ_2_, … with an asymptotically equivalent triangular array {δ_*n*1_,δ_*n*2_, … δ_*nn*_}. The original randomization scheme is applied within the array, except that treatment assignments are based on only the most recent *m* patients, with treatment assignments in earlier patients ignored. This makes the triangular array *m*-dependent [38], and *m* will be allowed to increase with *n*. Note that permuted-blocks randomization generates a sequence of δs that is already *m*-dependent, where *m* is the block size.

Because  it suffices to show the asymptotic normality of . It will first be shown that **δ′My**_0_ / *√n* is asymptotically normal and then that **δ′Mδ** / *n* → _p_*p*(1 − *p*), where → _p_ denotes convergence in probability.

As before, let **V** = Var(**δ**), with (*i*, *j*) element *v*_*ij*_. Make the following assumptions:

(A1)  as *n* → ∞.
(A2) Let (*a*_*n*1_, …, *a*_*nn*_)**′** = **a**_*n*_ = **M****y**_0_ and assume there is a finite *A* with | *a*_*ni*_ | < *A* for all *n* and *i*.
(A3) For all *i*, Σ_*j*_ | *v*_*ij*_ | → *B*_*i*_ as n → ∞, and there is a *B* with *B*_*i*_ < *B* for all *i*.

Let **δ**_*n*_ = (δ_*n*1_,δ_*n*2_, … δ_*nn*_)^***′***^, the vector of the *m*-dependent array elements, where *m* is selected as the smallest integer less than or equal to *n*^(0.5 − ϵ)^ for arbitrarily small ϵ > 0.

Now apply Berk's theorem [39]. His condition (i) on the uniform boundedness of *E* | *a*_*ni*_δ_*ni*_ | ^2 + *r*^ applies for arbitrarily large *r* owing to Assumption A2. His condition (ii) on the uniform boundedness of Var(*a*_*ni*_δ_*ni*_ + … + *a*_*nj*_δ_*nj*_) / (*j* − *i* + 1) for 1 *≤ i* < *j ≤ n* is satisfied because of both Assumptions A2 and A3. His condition (iii) on the limit of Var(*a*_*n*1_δ_*n*1_ + … + *a*_*nn*_δ_*nn*_) / *n* follows from Assumption A1. Finally, his condition (iv) on  is satisfied for *r* > (1 − ϵ) / ϵ, where ϵ was given above in the construction of the *m*-dependent array **δ**_*n*_. It follows that (*a*_*n*1_δ_*n*1_ + … + *a*_*nn*_δ_*nn*_) / *√n* converges in distribution to *N*(0,*v*).

Next, assume

(A4) the maximum eigenvalue of **V**, denoted *λ*_max_, is *o*(*n*).

Using the inequality **l**^***′***^**V****l***≤ λ*_max_ for any unit-length vector **l**, it is then straightforward to show that . First, let **M** = **I** − **L****L′**, where **L′L** = **I**_*q*_. Let **l**_*i*_ denote the *i*^th^ column of **L**, where without loss of generality, **l**_1_ = **1** / *√n*. Then

Now, , because *E*(**δ**^***′***^**1** / *n*) = 0 and . The result follows because for each *i* = 2, …, *q*,  and . Thus,  converges in distribution to *N*(0,*v* / {*p*(1 − *p*)}^2^).

Because *v* is unknown, practical application of the asymptotic normality of  for tests and confidence intervals requires a consistent estimator of *v* / {*p*(1 − *p*)}^2^. It will now be shown that  of Equation (A1.3) provides one solution, but a further assumption is needed.

(A5) *λ*_max_, the maximum eigenvalue of **V**, is *o*(*√n*). This is a stronger version of Assumption A4.

Now,

The pieces of this expression will be handled in turn, starting with the denominator. As shown above, . Next, with **M** = **I** − **L****L′** as above, . The trace of **V** is *np*(1 − *p*) and  is bounded between 0 and *qλ*_max_ / *n*, which converges to zero by A5. Thus, . Continuing with the denominator,  from both A5 and the convergence in probability of **δ′Mδ** / *n*.

For the numerator of ,  by Assumption A1. Next,  and it will be shown that , implying that . The following repeatedly uses the inequality **l′Vl***≤ λ*_max_ for any unit-length vector **l** and that  for any **x**.

which converges to 0 because of Assumptions A1 and A5. For the final term in the numerator of the expression,  converges in distribution to a central normal distribution, so in absolute value

which converges in probability to 0, completing the proof that  converges to a standard normal distribution.

## Appendix C

The dynamic randomization method of Section 5 that randomizes patients in a 2 to 1 ratio proceeds as follows. Determine the stratification variable values *s*_1_ and *s*_2_ for the current patient to be randomized and determine *n*_10_, *n*_11_, and *n*_12_ for previously randomized patients, where, for example, *n*_11_ equals the number of the previously randomized patients with Stratum 1 level equal to the level *s*_1_ of the current patient who were randomized to treatment level 1. Likewise, determine *n*_20_, *n*_21_, and *n*_22_. The assignment probabilities for the current patient are determined by the balance in these counts first for Stratum 1 and then for Stratum 2. If , then the treatment assignment probabilities depend on the pattern of the *n*_10_, *n*_11_, and *n*_12_ counts as follows:

- With three unique values, the probabilities are, respectively, 0.75, 0.15, and 0.10 for the treatments associated with the minimum, intermediate, and maximum treatment counts *n*_1*j*_
- With two values at the minimum count and one at the maximum, the probabilities are 0.40 for each of the treatments associated with the minimum count and 0.2 for the treatment associated with the maximum count
- With one value at the minimum count and two values at the maximum, the probabilities are 0.12 for each of the treatments associated with the maximum count and 0.76 for the treatment associated with the minimum count

If max(*n*_1*j*_) − min(*n*_1*j*_) = 0 then a similar check on Stratum 2 counts is performed, with unequal treatment assignment probabilities as above if there is imbalance. If there is balance of counts for Stratum 2, then the treatment for the current patient is selected from 0, 1, and 2 with equal probability.

To convert from 1 to 1 to 1 randomization to 2 to 1 randomization, patients randomized to treatment 0 are given control treatment and patients randomized to treatments 1 or 2 are given experimental.

The probabilities in the above algorithm were selected to match the conditional treatment assignment probabilities of the stratified PBR in blocks of six in the following sense. With complete randomization, the probability of assignment to experimental treatment is 2/3 regardless of previous treatment assignments, so the deviation from the target unconditional probability of 2/3 is zero. At the other extreme is a deterministic scheme where a fixed one-third of the patients are given control treatment and a fixed two-thirds are given experimental, so the squared deviation from the target unconditional probability of 2/3 is (0 − 2 / 3)^2^ / 3 + 2(1 − 2 / 3)^2^ / 3 = 0.22. In the example of Section 5 the mean squared deviation as estimated from the simulation is approximately 0.05 for both the stratified PBR and the sequential method described above.
